# Phylogenetic Analysis and Karyotype Evolution in Two Species of Core Gruiformes: *Aramides cajaneus* and *Psophia viridis*

**DOI:** 10.3390/genes11030307

**Published:** 2020-03-13

**Authors:** Ivanete de Oliveira Furo, Rafael Kretschmer, Patrícia C. M. O’Brien, Jorge C. Pereira, Malcolm A. Ferguson-Smith, Edivaldo Herculano Corrêa de Oliveira

**Affiliations:** 1 Post-Graduation Program in Genetics and Molecular Biology, Federal University of Pará, Belém, Pará 66075-110, Brazil; ivanetefuro100@gmail.com; 2 Laboratory of Tissue Culture and Cytogenetics, SAMAM, Evandro Chagas Institute, Ananindeua, Pará 67030-000, Brazil; 3 Cambridge Resource Centre for Comparative Genomics, Cambridge CB3 0ES, UK; rafa.kretschmer@gmail.com (R.K.); allsorter@gmail.com (P.C.M.O.); jorgecpereira599@gmail.com (J.C.P.); maf12@cam.ac.uk (M.A.F.-S.); 4 Pos-Graduation Program in Genetics and Molecular Biology, Federal University of Rio Grande do Sul, Porto Alegre, Rio Grande do Sul 91509-900, Brazil; 5 Faculty of Natural Sciences, Institute of Exact and Natural Sciences, Federal University of Pará, Belém, Pará 66075-110, Brazil

**Keywords:** Rallidae, Psophiidae, cytogenetic, chromosome evolution, phylogenetic

## Abstract

Gruiformes is a group with phylogenetic issues. Recent studies based on mitochondrial and genomic DNA have proposed the existence of a core Gruiformes, consisting of five families: Heliornithidae, Aramidae, Gruidae, Psophiidae and Rallidae. Karyotype studies on these species are still scarce, either by conventional staining or molecular cytogenetics. Due to this, this study aimed to analyze the karyotype of two species (*Aramides cajaneus* and *Psophia viridis*) belonging to families Rallidae and Psopiidae, respectively, by comparative chromosome painting. The results show that some chromosome rearrangements in this group have different origins, such as the association of GGA5/GGA7 in *A. cajaneus*, as well as the fission of GGA4p and association GGA6/GGA7, which place *P. viridis* close to *Fulica atra* and *Gallinula chloropus*. In addition, we conclude that the common ancestor of the core Gruiformes maintained the original syntenic groups found in the putative avian ancestral karyotype.

## 1. Introduction

Despite major progress in the reconstruction of phylogeny of Aves in the last decade, the classification of species within the order Gruiformes still represents one of the least stable among this class [1,2]. Nowadays, the order Gruiformes contains five modern families: Heliornithidae, Aramidae, Gruidae, Psophiidae and Rallidae, this last one greatly exceeding other Gruiformes families in species richness (144 species), geographical range and taxonomic complexity [3,4]. In addition, some gaps on the evolutionary history and interrelationships among Gruiformes species also remain unsolved. For example, the taxonomy of the Rallidae family has been the subject of debate, mainly because this group has adapted to similar environments across their geographic distribution, and consequently, they have been subject to convergence evolution, making difficult the understanding of their evolutionary origins [3,4,5]. Members of the Rallidae family inhabit a range of ecological environments, including freshwater and saltwater marshes, mangroves, sparsely vegetated atolls, cool-temperate woodlands, tropical forests and grasslands [3]. Similarly, the relationship of Psophiidae, a family of birds restricted to the Amazon basin forests, with the other five families included in the core Gruiformes is still controversial [4].

Concerning cytotaxonomic data, the karyotypes of Gruiformes are characterized by the typical avian formula, with diploid numbers (2n) close to 2n = 80, consisting of approximately 10 pairs of macrochromosomes and 30 pairs of indistinguishable microchromosomes [6,7,8]. However, there are species outside this standard, such as *Porzana albicollis* (2n = 72) [7] and *Fulica atra* (2n = 92) [6].

The advances in comparative chromosome mapping with the use of chromosome painting has provided important information for inferences about phylogenetic relationships in some groups of birds, clarifying some problems left by the analyses of molecular biology [9,10,11,12,13,14]. Furthermore, despite the apparent karyotypical conservation among birds observed by conventional staining, comparative chromosome painting has revealed many rearrangements, such as fusions and fissions in several macrochromosomes; this information allowed the inference of a putative ancestral karyotype (PAK) of birds, which is actually highly similar to the chromosomal complement of *Gallus gallus*, with the exception of pair 4, which corresponds to two distinct pairs in the proposed ancestral karyotype [9,10,11,15,16].

Up to now, comparative chromosome painting has been performed in only two species of Gruiformes—the coot (*Fulica atra—*FAT) and common moorhen (*Gallinula chloropus*—GCH), both belonging to the Rallidae family. *Fulica atra* (2n = 92) and *Gallinula chloropus* (2n = 78) share the fissions of the ancestral chromosomes GGA5 and GGA4 [17]. Moreover, these species also share chromosome associations between GGA4/5 and GGA6/7 [17]. However, in order to understand the dynamics of the karyotype evolution in this group, it is necessary to analyze other species belonging to different families of Gruiformes. 

Therefore, with the aim of broadening our understanding of the events occurring during the chromosomal evolution of Gruiformes, we carried out the comparative chromosome painting with chicken macrochromosome paints in two species of this order: *Aramides cajaneus*—ACA (Gray-necked Wood-Rail), a member of the Rallidae family and *Psophia viridis*—PVI (Green-winged Trumpeter), a member of the Psophiidae family. The goal of this study was to investigate (a) whether *Aramides cajaneus* shows a karyotype organization similar or different to *Fulica atra* and *Gallinula chloropus* and (b) whether *Psophia viridis* has similar or different rearrangements compared to Rallidade species. Based on the chromosome painting data for *Aramides cajaneus* and *Psophia viridis*, together with the data from the literature for other Gruiformes species, we discuss the possible process of karyotype evolution in Gruiformes.

## 2. Materials and Methods

### 2.1. Cell Cultures and Chromosome Preparations

Skin biopsies of *Aramides cajaneus* (one female) and *Psophia viridis* (one female) were collected at Museu Paraense Emilio Goeldi (Belém, PA, Brazil). The experiments were carried out according to the ethical protocols approved by an ethics committee (CEUA—Federal University of Pará) under no. 170/2013 and SISBIO 68443-1). Fibroblast cells were obtained from skin biopsies after dissociation with collagenase IV (0.0186 g in 4 mL of DMEM (Dulbecco’s Modified Eagle’s medium, Sigma-Aldrich, MO, USA), for 1 h at 37 °C, and maintained in DMEM medium (Sigma-Aldrich, MO, USA) supplemented with antibiotics (1%) and fetal bovine serum (15%) at 37 °C [18]. Chromosomal preparations were obtained after mitotic arrest by adding 100 µL colcemid (0.05 µg/mL) for 1 h, followed by suspension and incubation with 0.075 M KCl (10 min at 37 °C) and fixed in Carnoy’s fixative (3 methanol:1 acetic acid). Chromosome preparations were kept at −20 °C until the analyses.

### 2.2. Microscopic Analyses

At least 30 metaphases with conventional staining (Giemsa 5% in phosphate buffer, pH 6.8) were examined to determine the diploid number and chromosome morphology for each species. Images were captured using a 100× objective, microscopy DM1000 (Leica, CO, USA) and GenASIs software (ADS Biotec, Omaha, NE, USA) and the karyotype were ordered according to their arm ratios.

### 2.3. Chromosome Painting

Whole-chromosome probes of *G. gallus* (pairs 1–10) generated by flow-sorting (Cambridge Resource Centre for Comparative Genomics, Cambridge, UK) were labeled either with biotin or digoxygenin (Roche Diagnostics, Mannheim, Germany) by degenerate oligonucleotide-primed polymerase chain reaction (DOP-PCR) [19]. After denaturing in 70 °C for 10 min and preannealed for 30 min at 37 °C, the hybridization solution (1 µL labeled probe in 14 µL hybridization buffer) was added on slides with chromosome preparations previously desnatured at 70% formamide for 1 min and 20 s and dehydrated by serial ethanol dehydration (70%, 90% and 100%). Hybridization and detection by Avidin-Cy5 or anti-digoxygenin (Vector Laboratories, Burlingame, CA, USA), proceeded according to standard protocols [15]. Chromosomes were counterstained with 4′,6-diamidino-2-phenylindole (DAPI) (Sigma-Aldrich, St. Louis, MO, USA).

At least 10 metaphase spreads per individual were analyzed to confirm the hybridizations signals. FISH results were analyzed using a Zeiss Imager 2 microscope, 63× objective and images were captured using Axiovision v.4.8 software (Zeiss, Jena, Germany). Final edition of images was made using Adobe Photoshop CS6 software. For chromosomal evolution inferences, we used chromosome painting data from *Fulica atra* (FAT) and *Gallinula chloropus* (GCH) [17].

### 2.4. Phylogenetic Analysis

The inference of the phylogenetic tree was made based on cytogenetic information (chromosome painting) of four Gruiformes species, three belonging to the Rallidae Family (*Fulica atra*, *Gallinula chloropus* and *Aramides cajaneus*) and Psophiidae family (*Psophia viridis*), taking into consideration the presence or absence of chromosome features in these species.

## 3. Results

### 3.1. Karyotypes of Aramides cajaneus and Psophia viridis

**Figure 1:**
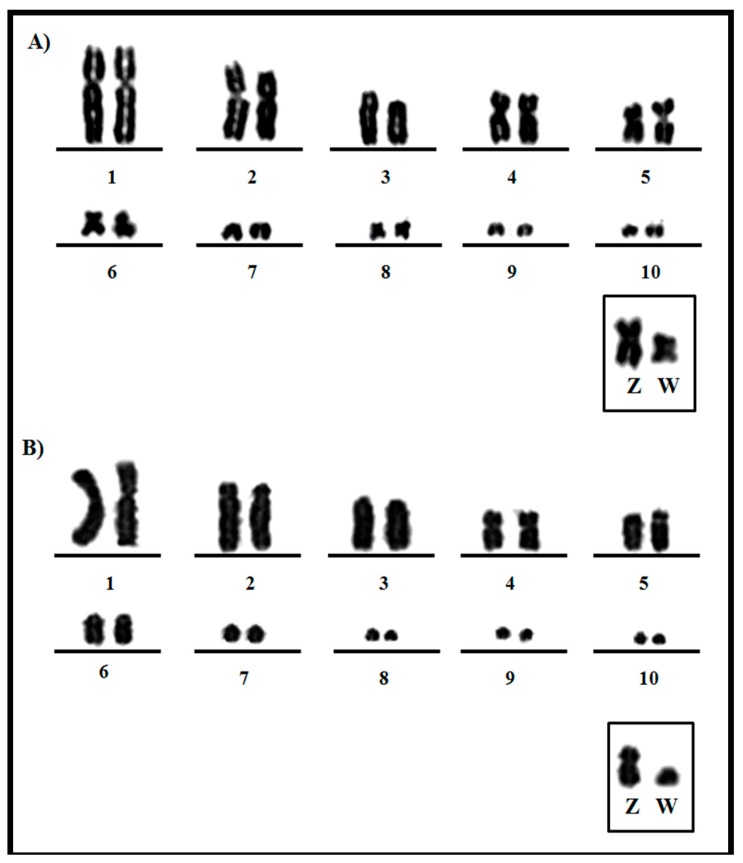
Partial karyotypes (pair 1–10 and ZW) of (**A**) *Aramides cajaneus* (2n = 78) and (**B**) *Psophia viridis* (2n = 80) (from pair 11 on, chromosomes correspond to indistinguishable microchromosomes, with the same size and morphology; for this reason, only the macrochromosomes are shown).

The karyotype of *Aramides cajaneus* has 2n = 78. Pairs 1, 2 and 4 are submetacentric, while 5, 6 and 8 are metacentric. The remaining autosomal chromosomes are telocentric (Figure 1A). The Z and W sex chromosomes are metacentric. *Psophia viridis* has a karyotype comprised of 80 chromosomes. Pair 1 is submetacentric, while pairs 2 and 5 are acrocentric, and pair 4 is metacentric and easily distinguishable from other chromosomes. The remaining autosomal chromosomes are telocentric. Among the sex chromosomes, Z is submetacentric and the W chromosome is telocentric (Figure 1B).

### 3.2. Chromosome Painting

**Figure 2:**
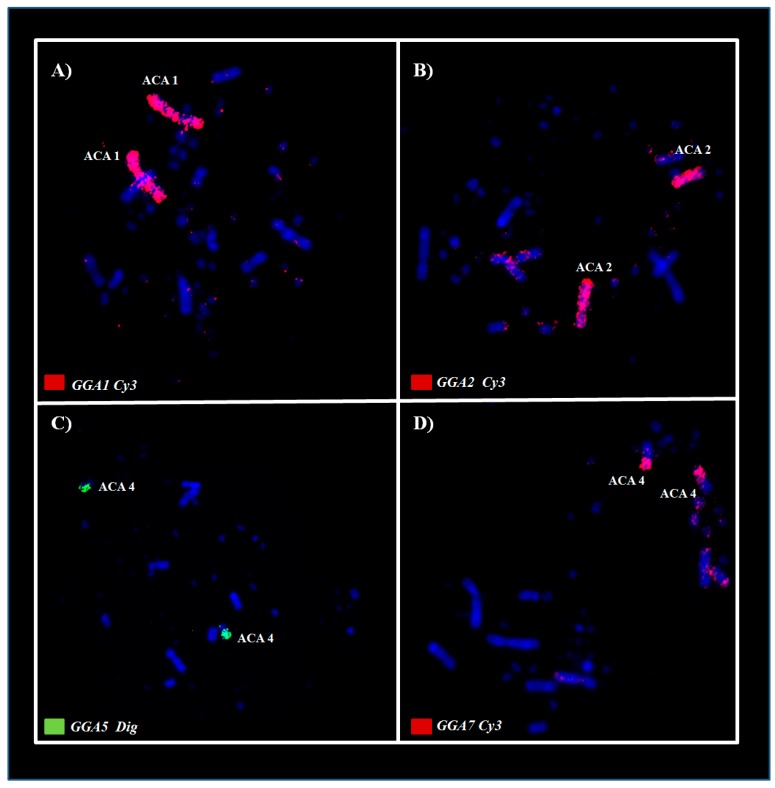
Experiments of chromosome painting using *G. gallus* probes in metaphases of *Aramides cajaneus*. (**A**,**B**) Examples of conserved syntenic groups (GGA1 and GGA2); (**C**,**D**) Examples of rearranged syntenic groups, showing an association between GGA5/ GGA7 on ACA4.

**Figure 3:**
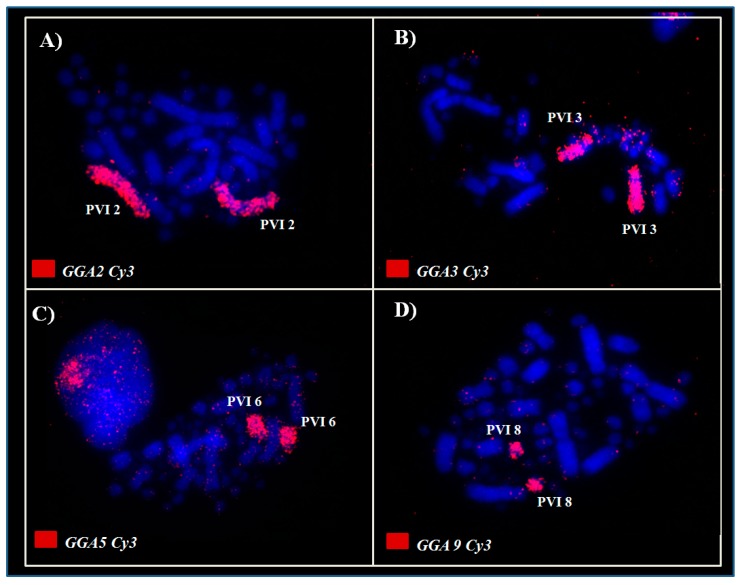
Representative examples of chromosome painting using macrochromosomes of *G. gallus* in *Psophia viridis*: GGA2 (**A**), GGA3 (**B**), GGA5 (**C**) and GGA 9 (**D**). The large pink fluorescence area on the top left corner of the (**C**) represent signals produced by the probes in interphase nucleus.

**Figure 4:**
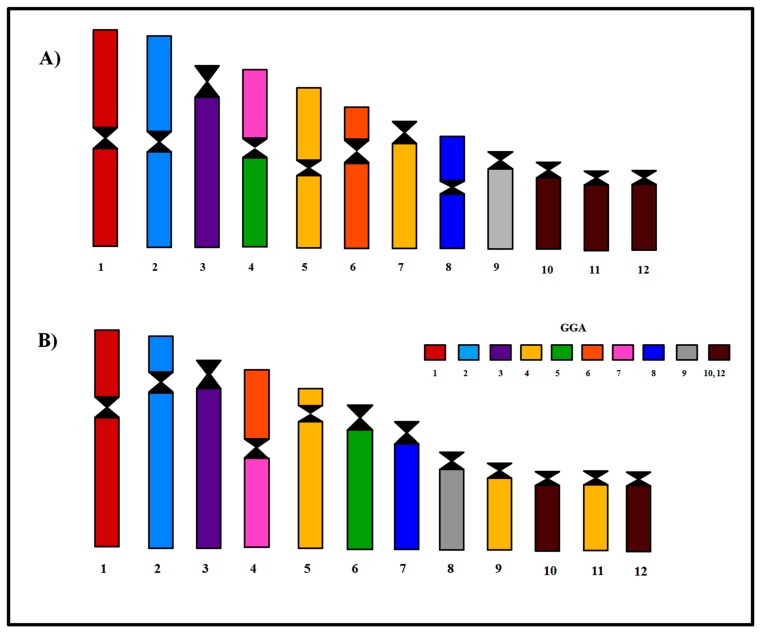
Homology maps with GGA probes in (**A**) *Aramides cajaneus* and (**B**) *Psophia viridis*.

The chicken probes corresponding to pairs GGA1–10 showed the following correspondence in the karyotype of *Aramides cajaneus* (ACA): GGA1 (ACA1); GGA2 (ACA2); GGA3 (ACA3); GGA4 (ACA5 and ACA7); GGA5 (ACA4q); GGA6 (ACA6); GGA7 (ACA4p); GGA8 (ACA8); GGA9 (ACA9) and GGA10 (ACA10). In addition, an association between GGA5/GGA7 was identified in (ACA4) (Figure 2C,D); therefore, a total of 11 homology signals were found in the karyotype of *A. cajaneus* with GGA probes (Figure 2 and Figure 4A). However, in *Psophia viridis*, chicken painting probes showed a slightly different correspondence when compared to *Aramides cajaneus*. Hence, the homologies between chicken and *Psophia viridis* (PVI) are: GGA1 (PVI1); GGA2 (PVI2); GGA3 (PVI3); GGA4 (PVI5, PVI9 and ACA11); GGA5 (PVI6); GGA6 (PVI4q); GGA7 (PVI4p); GGA8 (PVI7); GGA9 (PVI8) and GGA10 (PVI9). Furthermore, a fission in GGA4 and an association between GGA6/GGA7 in (PVI4) were observe in this species, thereby, a total of 12 homology signals were observed between the chromosomes of *P. viridis* and GGA probes (Figure 3; Figure 4B).

### 3.3. Syntenic Blocks Shared among Gruiformes Species and Phylogentic Analyses

**Figure 5:**
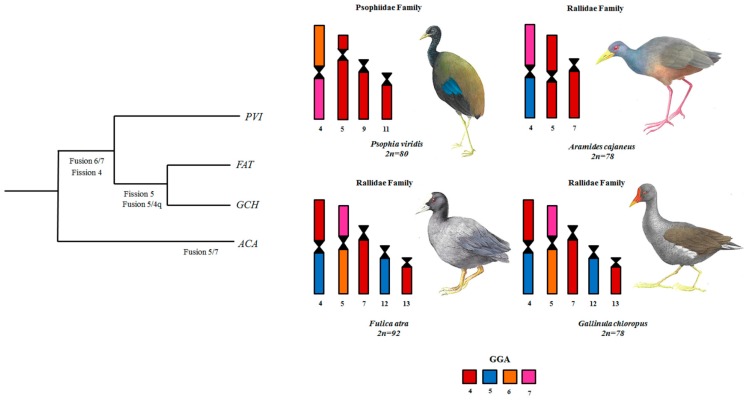
Schematic representation of chromosome rearrangements during evolution of the Gruiformes based on comparative chromosome painting and literature results (Nanda et al., 2011). We propose that the *A. cajaneus* would be more basal within the Rallidae family and *P. viridis* close to *F. atra* and *G. chloropus*. Legend: *Fulica atra* (FAT), *Aramides cajaneus* (ACA), *Gallinula chloropus* (GCH), *Psophia viridis* (PVI), *Gallus gallus* (GGA).

In order to proceed with the phylogenetic analysis, comparative chromosome painting data covering the homology of macrochromosome pairs of four species of Gruiformes—*Aramides cajaneus* and *Psophia viridis* from the present work, and *Fulica atra* and *Gallinula chloropus* previously described by [17]—were organized in a matrix (Table 1). The phylogenetic tree obtained is shown in Figure 5. *F. atra* and *G*. *chloropus* are more derived in relation to the *A. cajaneus*, and form a clade supported by three rearrangements—the fusion GGA4/GGA5 and GGA6/GGA7, and also the fission of GGA4p—chromosome features not observed in *A. cajaneus*. Concerning *P. viridis,* it would be closer to the clade formed by *F. atra* and *G*. *chloropus*, with which this species shares two rearrangements: the fission of GGA4p and the association GGA6/GGA7.

## 4. Discussion

The diploid chromosome numbers of both species analyzed are very similar, *A. cajaneus* 2n = 78 and *P. viridis* 2n = 80 (Figure 1). Additionally, both species are characterized by a typical avian karyotype, since the mode of the chromosome number in birds is 2n = 80, with a high number of microchromosomes [16,20].

In general, rearrangements found in Gruiformes involved chromosome pairs homologous to GGA4, GGA5, GGA6 and GGA7. The fission of ancestral syntenies GGA4p and GGA5 followed by fusion have been proposed as ancestral events in Gruiformes, since these rearrangements are present in *F. atra* and *G. chloropus* [17]. However, in *P. viridis,* only the fission in GGA4p has been observed, without the fusion between GGA4q/GGA5 (Figure 4A). Furthermore, *A. cajaneus* does not share these rearrangements (Figure 5).

The association GGA6/GGA7 has been found in two species of family Rallidae (*F. atra* and *G. chloropus*) [17] and family Psophiidae (*P. viridis*); however, it is absent in *A. cajaneus* (Table 1). The association between GGA6/GGA7 has been detected in five species belonging to different orders of birds: Galliformes—*Numida meleagris* [21], Strigiformes—*Pulsatrix perspicillata* [22], Trogoniformes—*Trogon s. surrucura* [23], Psittaciformes—*Nymphicus hollandicus*, *Agapornis roseicollis*, *Melopsittacus undulates* [24], *Ara macao* [25], *Ara chloropterus*, *Anodorhynchus hyacinthinus* [9], *Psittacus erithacus* [26], *Pyrrhura frontalis*, *Amazona aestiva* [11] and Columbiformes—*Leptotila verreauxi* [16]. Hence, GGA6/GGA7 association originated independently in five different orders.

The apparent multiple independent origins of associations between GGA6/GGA7 in avian species suggest that there are specific sites in these chromosomes that are susceptible to rearrangement processes (hotspots) [27,28,29]. This fact highlights the potential for the occurrence of rearrangements, such as chromosomal fusions, inversions and centromere shifts [30].

### Phylogenetic Analysis

The results obtained from the experiments in this study, together with data previously published concerning two other Gruiformes (Table 1; Table 2), were plotted using the phylogenetic tree proposed by [4] and [5] to improve understanding of chromosomal evolution in this order (Figure 5).

In respect of family Rallidae, some phylogenetic relationships, such as *F. atra* and *G. chloropus* as sister-groups, are well supported [31,32]. According to [5], *A. cajaneus* was included in the ‘‘Aramides clade”, as a sister-group of “Fulica clade”. The chromosome data of these species corroborate this relationship, since *F. atra* and *G. chloropus* share the same chromosome rearrangements, such as associations GGA6/GGA7, GGA4/GGA5 and fissions of GGA4p and GGA5, not found in *A. cajaneus* (Figure 4B and Table 1 and Table 2). In this way *A. cajaneus*, which shares less chromosome syntenies with the other two species (*F. atra* and *G. chloropus*), would be more basal than the ‘Fulica clade’.

In our previous study with *Eurypyga helias* (Eurypygidae), formerly included in Gruiformes, but now regarded as belonging to a different order (Eurypygiformes) with only two families (Rynochetidae and Eurypygidae), we had proposed that the putative common ancestral karyotype of core Gruiformes would have a fission of GGA4p, since it had been found in the three species studied (*P. viridis*, *F. atra* and *G. chloropus*) (Table 1; Table 2) [10,17]. If this hypothesis was correct, *A. cajaneus* would have regained the ancestral character. Alternatively, the absence of this fission in *A. cajaneus* indicates that it does not represent a synapomorphy of the Gruiformes.

According to [29], there are regions of bird genomes that are prone to breakage, facilitating chromosomal rearrangements, and this could explain the fission of GGA4p in some species but not in *A. cajaneus*. As more bird genomes are sequenced, the reasons for the conservation of some syntenic groups, while others were disrupted and reorganized, will become clearer.

Despite the fact that the phylogenetic position of the Psophiidae family is not well resolved within the Core Gruiformes [4], our results show that *P. viridis* shares some rearrangements with *F. atra* and *G. chloropus*, such as fission of GGA4p and association of GGA6/GGA7 (Table 1; Table 2); these rearrangements place *P. viridis* close to *F. atra* and *G. chloropus* (Rallidae family) (Figure 5).

Accordingly, the last common ancestor from the Core Gruiformes would have a karyotype similar to the putative ancestral avian karyotype (PAK) [16], because some rearrangements that were found in this group are the result of independent events, such as the association between GGA5/GGA7 in *A. cajaneus*, despite the fact that some association and fission events were not found in all the species analyzed (Figure 5).

Thus, molecular cytogenetic analysis confirms earlier studies on the relationships between *F. atra* and *G. chloropus*. Furthermore, they support the fact that the chromosome rearrangements accumulated in *P. viridis* are similar to the Rallidae species, although *A. cajaneus* shows a karyotype organization different from *F. atra* and *G. chloropus*. Nevertheless, this study has improved our understanding of the process of karyotype evolution in the Core Gruiformes.

## Figures and Tables

**Table 1:** Chromosomal homologies among Gruiformes species and *Gallus gallus* (GGA1-10).

Chicken Chromosome Paint Number	*F. atra*, FAT, 2n = 92 [17]	*G. chloropus*, GCH, 2n = 78 [17]	*A. cajaneus*, ACA, 2n = 78 (Present Study)	*P. viridis*, PVI, 2n = 80 (Present Study)
GGA1	FAT1	GCH1	ACA1	PVI1
GGA2	FAT2	GCH2	ACA2	PVI2
GGA3	FAT3	GCH3	ACA3	PVI3
GGA4q	FAT4p	GCH4p	ACA5	PVI5
GGA5	FAT4q, FAT12	GCH4q, GCH12	ACA4q	PVI6
GGA6	FAT5q	GCH5q	ACA6	PVI4q
GGA7	FAT5p	GCH5p	ACA4p	PVI4p
GGA8	FAT6	GCH6	ACA8	PVI7
GGA9	FAT8	GCH8	ACA9	PVI8
GGA4p	FAT7, FAT13	GCH7, GCH13	ACA7	PVI9, PVI11
GGA10	FAT9	GCH9	ACA10	PVI9

**Table 2:** Chromosomal rearrangements observed in Gruiformes, according to comparative chromosome painting with *G. gallus* probes.

Family	Species	Rearrangements (GGA)					References
		Associations		Fission			
		GGA6/7	GGA5/7	GGA4 (2 pairs)	GGA4 (3 pairs)	GGA5	
Psophiidae	*Psophia viridis*	*			*		Present study
Rallidae	*Aramides cajaneus*		*	*			Present study
Rallidae	*Fulica atra*	*			*	*	[17]
Rallidae	*Gallinula chloropus*	*			*	*	[17]

The presence of the rearrangement is indicated by *.
